# Genetic structure and isolation by altitude in rice landraces of Yunnan, China revealed by nucleotide and microsatellite marker polymorphisms

**DOI:** 10.1371/journal.pone.0175731

**Published:** 2017-04-19

**Authors:** Di Cui, Cuifeng Tang, Jinmei Li, Xinxiang A, Tengqiong Yu, Xiaoding Ma, Enlai Zhang, Yanjie Wang, Guilan Cao, Furong Xu, Luyuan Dai, Longzhi Han, Hee-Jong Koh

**Affiliations:** 1 Institute of Crop Science, Chinese Academy of Agricultural Sciences, Key Laboratory of Crop Germplasm Resources and Utilization, The National Key Facility for Crop Gene Resources and Genetic Improvement, Ministry of Agriculture, Beijing, China; 2 Department of Plant Science, Research Institute for Agriculture and Life Science, Seoul National University, Seoul, Republic of Korea; 3 Institute of Biotech and Germplasm Resources, Yunnan Academy of Agricultural Sciences, Kunming, Yunnan, China; 4 Plant Genomics and Breeding Institute, Seoul National University, Seoul, Republic of Korea; National Cheng Kung University, TAIWAN

## Abstract

Rice landraces, a genetic reservoir for varietal improvement, are developed by farmers through artificial selection during the long-term domestication process. To efficiently conserve, manage, and use such germplasm resources, an understanding of the genetic structure and differentiation of local rice landraces is required. In this study, we analyzed 188 accessions of rice landraces collected from localities across an altitudinal gradient from 425 to 2, 274 m above sea level in Yunnan Province, China using ten target genes and 48 SSR markers. We detected clear differentiation of the rice landraces into *indica* and *japonica* groups and further separation of the accessions in each group into two subgroups according to altitude, including a lower altitude subgroup and higher altitude subgroup. The AMOVA results showed significant genetic differentiation among altitude zones at SSRs and most genes, except *Os1977* and *STS22*. We further determined that differentiation among landrace populations followed a model of isolation by altitude, in which gene flow was higher among populations at similar altitude levels than across different altitude levels. Our findings demonstrated that both adaptation to altitude and altitude-dependent gene flow played key roles in the genetic differentiation of rice landraces in Yunnan, China.

## Introduction

Crop domestication is a complex process mediated by a series of phenotypic changes to improve cultivation, harvesting, and consumption. Rice (*Oryza sativa* L.) is one of the earliest domesticated crop species, and the genetic diversity of cultivated rice has been reduced by up to 80% from that of the wild ancestor during the domestication and artificial selection processes [1]. The most extreme loss of diversity is found in modern high-yielding rice varieties, and this has serious consequences for disease susceptibility and adaptation to changing environments [2]. By contrast, rice landraces, which originated and evolved in the field over millennia via selective breeding by farmers, have retained genetic variation [3]. This variation has important implications in rice breeding by providing new genes/alleles for crop improvement. However, with the development of modern agriculture, a large number of local landraces have been replaced with modern varieties introduced over the past 40 years [4]. In China, rice landraces are no longer planted in most provinces, with the exception of some ethnic minority regions, such as areas of Yunnan and Guizhou Provinces.

Selection by various ethnic groups inhabiting areas of different altitudes and climatic conditions and with different cultivation methods, cultures, and traditions has contributed to rice crop diversity in Yunnan. Accordingly, Yunnan is one of the largest centers of genetic diversity for rice worldwide [5–7]. Rice landraces in Yunnan are widely distributed in a region from 21°8′ 32′′ N to 29°11′ 18′′ N and 97°31′ 39′′ E to 106°11′ 47′′ E, and are planted at various altitudes and under diverse climatic conditions [7]. Landraces grow from altitudes of approximately 76 m in Hekou county, Honghe prefecture in the southeastern part of Yunnan to 2,700 m in Weixi county, Diqing prefecture [7]. The wide distribution of rice landraces provides an excellent opportunity for studies of genetic structure and differentiation patterns of rice landraces along the altitudinal range as well as the role of altitude in shaping population genetic structure. Several studies have examined genetic differentiation and the distribution of rice landraces along altitudinal gradients in Yunnan based on phenotype traits or insertion/deletion (indel) molecular markers [8–10]. According to a previous study, both *indica* and *japonica* rice varieties are cultivated in Yunnan [6], and the distribution of rice landraces can be artificially categorized into three rice cultivation regions as follows: (1) the *indica* belt at altitudes of below 1,400 m, (2) the mixed *indica* and *japonica* belt at altitudes of between 1,400 and 1,600 m, and (3) the *japonica* belt at altitudes of above 1,600 m [8]. However, little is known about how altitudinal variation affects population genetic structure. Furthermore, it is not clear whether rice landraces in Yunnan exhibit isolation by altitude. These questions need to be explored using DNA data.

Among various molecular marker types, simple sequence repeat (SSR) markers are commonly used to estimate genetic diversity, population structure, and differentiation in numerous plant species [11]. Furthermore, with the development of DNA sequencing technology, multi-locus DNA sequences have been successfully used to estimate genetic diversity and for phylogenetic analyses [12–15]. DNA sequence differences directly reflect genetic differences; accordingly, sequence comparison is an ideal method for revealing genetic diversity and differentiation.

We collected diverse rice landraces from Yunnan, China along a wide range of altitudes and analyzed gene sequences and SSR markers. Our objectives were to examine the genetic structure of rice landraces and to assess the role of isolation by altitude in genetic differentiation.

## Materials and methods

### Ethics statement

Field work and the collection of leaves were approved by Institute of Crop Science, CAAS (Chinese Academy of Agricultural Sciences). In this study, the land accessed is not privately owned or protected, and any protected or endangered species were not sampled.

### Sampling and choice of loci

In total, 188 rice landraces were collected from a wide altitudinal range (425–2,274 m above sea level) in Yunnan Province, China (S1 Table). These rice landraces were artificially divided into eight groups according to altitude at intervals of 200 m, except for the lowest (≤ 800 m) and highest (≧2,000) zones (S2 Table). The geographic localities of the rice landraces sampled in this study are shown in S1 Fig. In addition, two varieties, 93–11 and Nipponbare, were used as references for *indica* and *japonica*, respectively.

A set of 48 SSRs evenly distributed throughout the rice genome (S3 Table) and ten unlinked nuclear gene loci were used in this study. Five of the genes, *CatA*, *GBSSII*, *Os1977*, *STS22*, and *STS90*, which have been used to estimate nucleotide diversity in rice populations in previous studies, were used [16,17]. Five additional genes, *Ehd1*, *S5*, *Pid3*, *GS3*, and *GS5*, which are associated with agronomic traits in rice, were also used [18–22]. Schematic diagrams of all ten genes are shown in S2 Fig. Detailed information about the genomic location and putative functions of the genes as well as the primer sequences for amplification can be found in S4 Table.

### DNA extraction, SSR genotyping, and gene sequencing

Total genomic DNA was extracted from fresh seedling leaves using a modified CTAB procedure [23]. A total of 48 SSRs were amplified by polymerase chain reaction (PCR) with fluorescently labeled primers in a 10-μL reaction volume containing 20 ng of genomic DNA, 10× PCR reaction buffer, 10 mM Mixture dNTP, 2 μM primers, and 0.5 units of Taq polymerase. The PCR profile was as follows: pre-denaturation at 94°C for 5 min, 36 cycles of denaturation at 94°C for 30 s, annealing at 55–60°C (dependent on primers) for 30 s, and extension at 72°C for 40 s, and a final extension at 72°C for 10 min. PCR products were size separated on a 3730XL DNA Sequencer equipped with GENESCAN software (ABI, Waltham, MA, USA). Fragment size was recorded using Gene Marker V1.6 (SoftGene, State College, PA, USA) and manually re-checked.

For the detection of genes, PCR was performed in a 25-μL volume consisting of 0.2 μM of each primer, 200 μM of each dNTP, 10 mM Tris—HCl (pH 8.3), 50 mM KCl, 1.5 mM MgCl_2_, 0.5 U of HiFi DNA polymerase (Transgen, Beijing, China), and 10–30 ng of genomic DNA. The PCR profile was as follows: pre-denaturation at 94°C for 5 min, 36 cycles of denaturation at 94°C for 30 s, annealing at 55–60°C (dependent on primers) for 30 s, and extension at 72°C for 1.5 min, and a final extension at 72°C for 10 min. The PCR products were electrophoresed on 1.2% agarose gels, and DNA fragments were cut from the gel and purified using a Tiangen Gel Extraction Kit (Tiangen, Beijing, China). Sequencing reactions were performed using an ABI 3730 Automated Sequencer. Initially, all samples were directly sequenced. However, if haplotypes could not be readily inferred owing to heterozygosity, the PCR product was ligated into an EASY vector (Transgen) and at least four clones were sequenced. For heterozygous individuals, one allele sequence was randomly selected. Because Taq errors occurred, when polymorphisms were only found in one accession, this accession was re-sequenced with the cloning step to verify the polymorphisms.

### Population genetic structure

To identify population structure, a Bayesian clustering analysis was conducted using STRUCTURE 2.2 [24,25] based on the 48 SSRs and SNP data, respectively. Fifteen independent runs were performed for each *k* value (from 1 to 12), using a burn-in length of 100,000, a run length of 100,000, and admixture and correlated allele frequency models. The *k* value was determined based on LnP(D) in the STRUCTURE output and the ad-hoc statistic Δ*k* [26,27]. A principal component analysis (PCA) was performed using NTSYSpc version 2.11 [28] based on SSR data.

### Analysis of DNA sequence and SSR data

DNA sequences were aligned using ClustalX 1.83 [29] and edited using BioEdit 7.0.9.0 [30]. Indels were not included in the analysis. For each locus, the number of segregating sites (S), the number of haplotypes (h), haplotype diversity (Hd), and two nucleotide diversity parameters, mean pairwise differences (*θ*_π_) [31] and Watterson’s estimator based on the number of segregating sites (*θ*_w_) [32], were determined using DnaSP version 5.0 [33]. The minimum number of recombination events (Rm) was estimated using the four-gamete test [34]. The statistical analysis of SSR data, including allele number, genotype number, number of private alleles (i.e., alleles that only appear in certain populations), heterozygosity, gene diversity, and polymorphism information content (PIC), was implemented in PowerMarker version 3.25 [35].

### Haplotype network analysis

Haplotype networks were constructed based on mutational steps using Network 4.5 [36]. These networks represent the genetic distances among DNA sequences or alleles and are represented by circles of different sizes and colors and lines linking the circles. Because many haplotypes were obtained for the nuclear loci, only major haplotypes, i.e., those observed in more than three individuals, were selected for network construction.

### Genetic differentiation and isolation by altitude

The overall distribution of nucleotide diversity was investigated using an analysis of molecular variance (AMOVA) implemented in Arlequin 3.01 [37]. Sequence variation was hierarchically partitioned between the two subpopulations, among altitude zones within subpopulations, and within altitude zones. The significance of all estimated fixation indices was tested using 10,000 permutations, as described by Excoffier et al. [37]. Pairwise *F*_ST_, generally expressed as the proportion of genetic diversity explained by allele frequency differences among populations [38], was used to measure differentiation within and between subpopulations, as implemented in Arlequin 3.01 [37]. Isolation by altitude was evaluated by assessing the correlation matrix between pairwise altitude difference and genetic differentiation between altitude zones was assessed using Mantel’s tests implemented in Arlequin 3.01 [37]. A total of 10,000 random permutations were performed.

## Results

### Population structure and genetic relationship analyses

**Fig 1:**
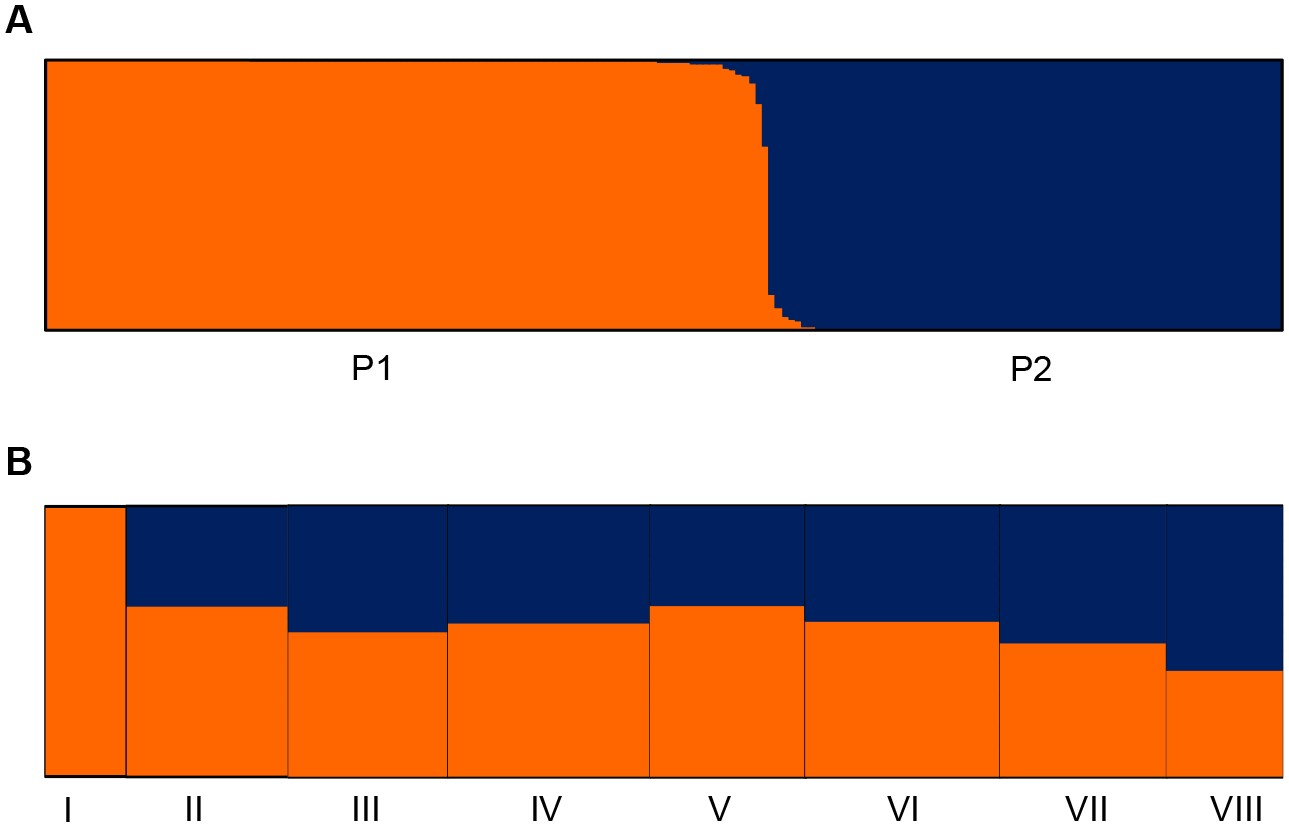
Model-based ancestries and their distribution in altitude zones. (A) Model-based ancestry of each accession in P1 and P2; (B) distribution of model-based populations in each altitude zone.

**Fig 2:**
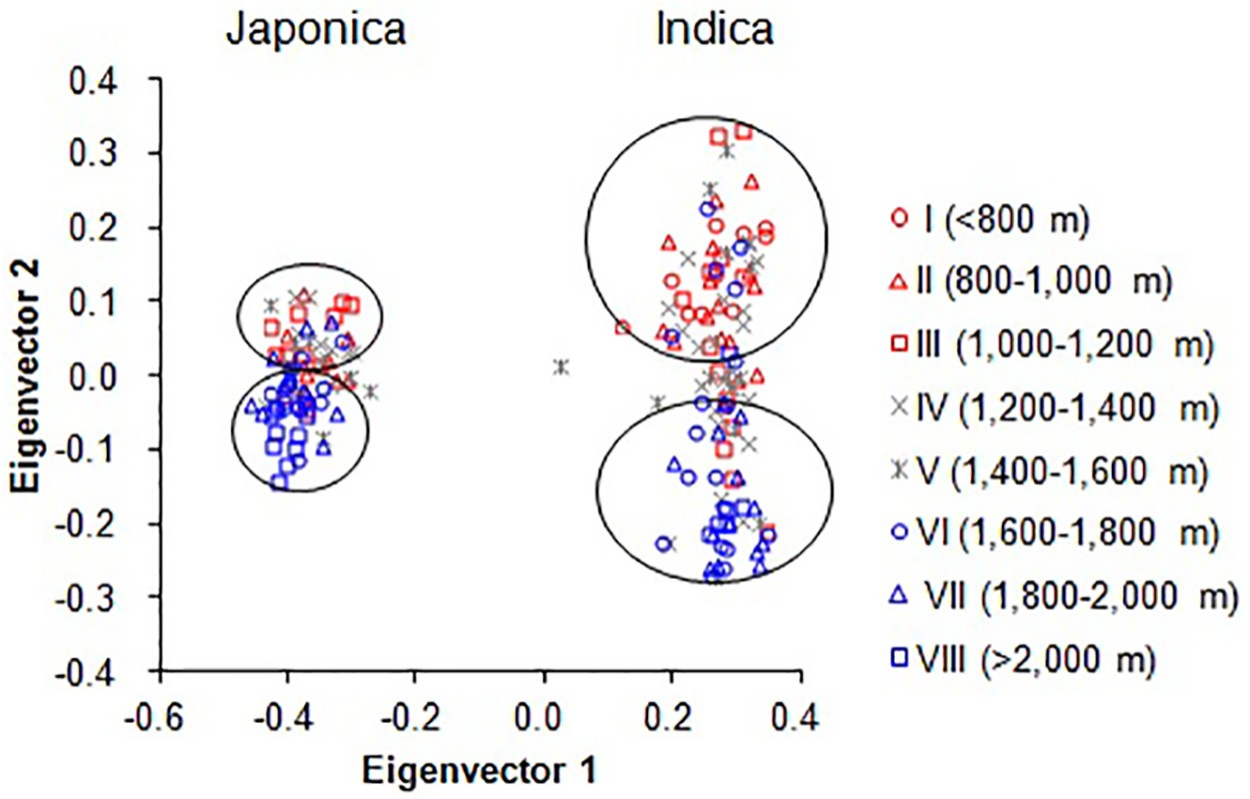
Principal component analysis of rice landraces from eight altitude zones.

To infer the population structure of 188 rice landraces, we performed model-based simulations using 48 SSRs and 89 SNPs from ten genes, respectively. We observed an increase in the mean posterior probability LnP(D) as the number of groups *k* increased, but we detected a sharp peak of Δ*k* at *k* = 2 (S3A Fig) for rice landraces using 48 SSRs. These results were also supported by structural analysis based on SNP data (S3B Fig). These results suggest that rice landraces could be grouped into two subpopulations, referred to as P1 and P2 (Fig 1A). Based on the reference cultivars, the 110 P1 accessions were *indica* or *indica*-like, and the 78 P2 accessions were *japonica* or *japonica*-like. This result demonstrated that rice landraces from Yunnan were clearly differentiated into *indica* and *japonica* groups. Fig 1B shows the distribution of rice accessions from the two subpopulations in each altitude zone. We found only *indica* at altitudes below 800 m, but we observed both *indica* and *japonica* in all other altitude zones. We detected a significant negative correlation between the proportion of *indica* rice landraces in various altitude zones and altitude (*r* = -0.814, P < 0.05), but a significant positive correlation between the proportion of *japonica* rice landraces in various altitude zones and altitude (*r* = 0.814, P < 0.05) (S4 Fig). In other words, the proportion of rice landraces classified as *indica* in each altitude zone in Yunnan decreased from low to high altitudes. By contrast, the proportion of landraces classified as *japonica* in each altitude zone increased from low to high altitudes. Based on a PCA (Fig 2), all rice landraces were distinctly divided into two main groups, *indica* and *japonica*, supporting the population structure revealed by STRUCTURE. In each group, we detected further separation into two subgroups according to altitude, including a lower altitude subgroup and higher altitude subgroup, with a few exceptions in middle altitude zones. The *indica* group was more highly dispersed than the *japonica* group, indicating higher diversity based on SSRs.

### Nucleotide diversity in model-based populations

We sequenced ten unlinked loci from 188 Yunnan rice landraces covering a wide altitudinal gradient. The aligned sequences for each locus ranged from 420 to 627 bp, with a total length of 4,994 bp, including 1120 bp of coding sequence (S4 Table). We excluded 17 indel polymorphisms, ranging from 1 to 5 bp, from the data analyses. Standard sequence polymorphism statistics at each locus are summarized in Table 1. We observed genetic diversity, as estimated by *θ*_w_, ranging from 0.0014 (*STS22*) to 0.0082 (*CatA*) for rice accessions from the *indica* subpopulation and from 0.0010 (*S5*) to 0.0074 (*CatA*) for accessions from the *japonica* subpopulation. We did not observe a significant difference between the average nucleotide diversity at silent sites between *indica* accessions (*θ*_π_ = 0.0034, *θ*_w_ = 0.0030) and the *japonica* subpopulation (*θ*_π_ = 0.0032, *θ*_w_ = 0.0031; *P* > 0.05 for both *θ*_π_ and *θ*_w_). In general, based on SSR markers, we detected more genetic variation (gene diversity and PIC), but less heterozygosity, in *indica* rice than in *japonica* rice (S5 Table).

**Table 1:** Summary of nucleotide polymorphisms.

Population	Locus	S	*h*	Hd	*θ*_π_	*θ*_w_
P1 (*Indica*)	*CatA*	18	11	0.515	0.0106	0.0082
	*GBSSII*	4	4	0.456	0.0032	0.0018
	*Os1977*	4	2	0.019	0.0002	0.0018
	*STS22*	4	5	0.67	0.0018	0.0014
	*STS90*	8	7	0.725	0.0039	0.0032
	*S5*	6	4	0.438	0.0032	0.0018
	*Pid3*	6	5	0.175	0.0010	0.0024
	*Ehd1*	12	11	0.465	0.0027	0.0049
	*GS3*	6	13	0.688	0.0036	0.0020
	*GS5*	7	12	0.415	0.004	0.0024
	Average	7.5	7.4	0.4566	0.0034	0.0030
P2 (*Japonica*)	*CatA*	15	5	0.184	0.0012	0.0074
	*GBSSII*	3	3	0.249	0.0017	0.0015
	*Os1977*	4	3	0.149	0.0009	0.0020
	*STS22*	4	6	0.754	0.0021	0.0015
	*STS90*	8	8	0.65	0.0051	0.0036
	*S5*	3	3	0.123	0.0004	0.0010
	*Pid3*	7	7	0.606	0.0041	0.0030
	*Ehd1*	10	6	0.412	0.0072	0.0044
	*GS3*	5	7	0.546	0.0014	0.0018
	*GS5*	14	13	0.723	0.0075	0.0051
	Average	7.3	6.1	0.4396	0.0032	0.0031

S, number of segregating sites; *h*, number of haplotypes; Hd, haplotype diversity; *θ*_π_, nucleotide diversity; *θ*_w_, Watterson’s parameter for silent sites.

### Phylogenetic and geographic analyses of haplotypes

**Fig 3:**
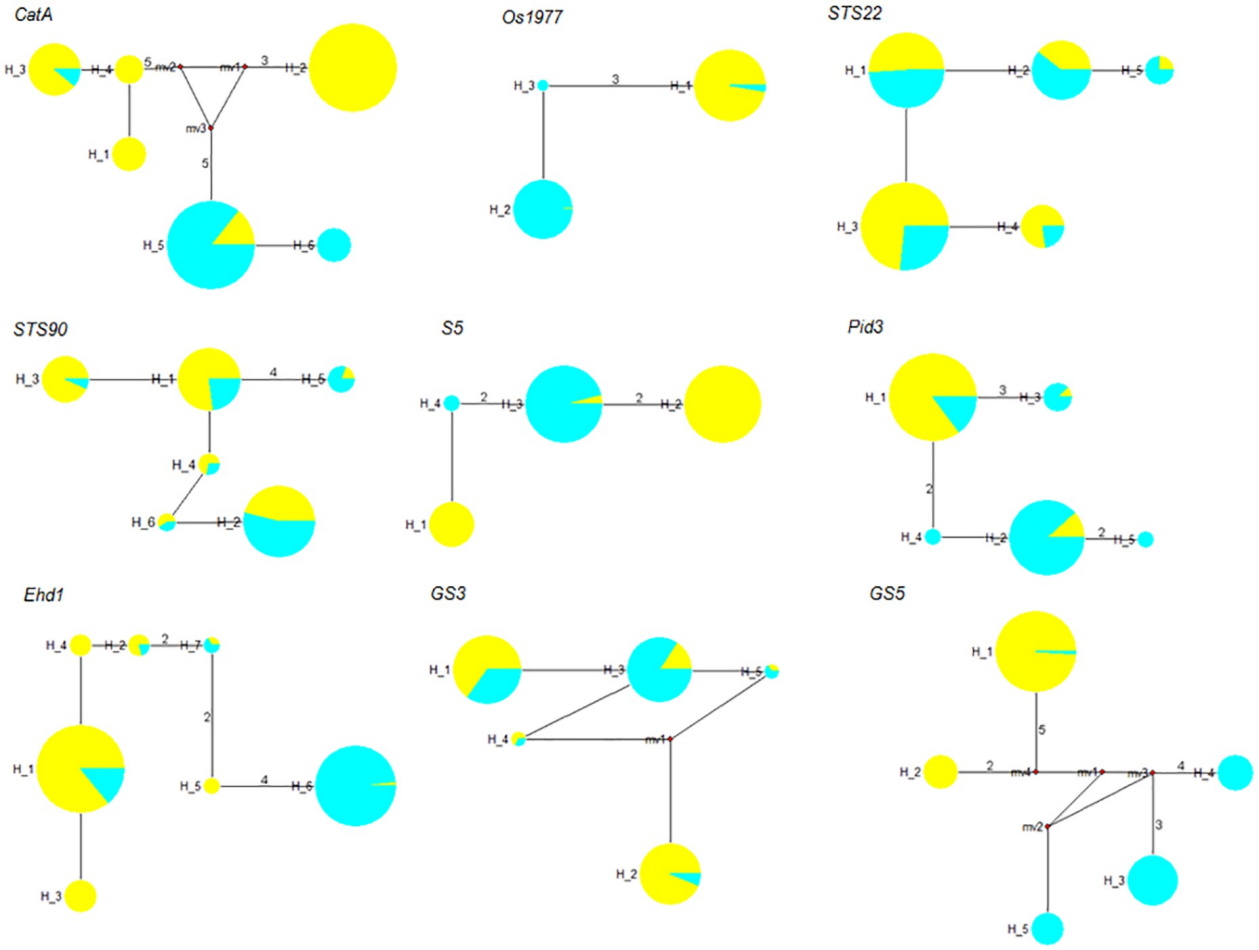
Haplotype networks for each gene. The circle size is proportional to the quantity of samples with a given haplotype, and the numbers next to the circles represent the haplotype number. Lines between haplotypes represent mutational steps between alleles. When more than one nucleotide difference existed between linked haplotypes, the number is indicated next to the lines. Colors for rice landraces collected from different subpopulations are as follows: yellow, P1 (*indica*) subpopulation; blue, P2 (*japonica*) subpopulation. The haplotype network for *GBSSII*, which had only two haplotypes, is not shown.

Fig 3 shows haplotype networks constructed based on the major haplotypes for each gene. For *CatA*, *GBSSII*, *Os1977*, *STS22*, *STS90*, *S5*, *Pid3*, *Ehd1*, *GS3*, and *GS5*, we observed 6 (5 and 3 for *indica* and *japonica* accessions), 2 (2 and 2), 3 (1 and 3), 5 (5 and 5), 6 (6 and 6), 4 (3 and 2), 5 (3 and 5), 7 (7 and 4), 5 (5 and 5), and 5 (2 and 4) haplotypes, respectively. In total, we found ten *indica*-specific haplotypes in *CatA*, *S5*, *GS5* and *Ehd1*, and eight *japonica*-specific haplotypes in *CatA*, *Os1977*, *Pid3*, *S5* and *GS5*. In addition, we detected distinct differences in haplotype frequency between *indica* and *japonica* rice landraces. We detected five haplotypes (H_3 of *CatA*; H_2 of *GS3*; H_1 of *GS5*; H_1 of *Os1977*; H_3 of *STS90*) that were common in *indica* rice, with an average frequency of 52.51% (8.16–99.05%), but rare in *japonica* rice, with an average frequency of 2.82% (1.43–4.05%). We observed six haplotypes (H_6 of *Ehd1*; H_3 of *Pid3*; H_2 of *Os1977*; H_5 of *STS22*; H_3 of *S5*; H_5 of *STS90*) that were rare in *indica* rice, with an average frequency of 1.55% (0.95–2.78%), but were more frequent in *japonica* rice, with an average frequency of 49.22% (8.00–94.81%). These results further indicated that there was obvious genetic differentiation between *indica* and *japonica* rice landraces at some loci.

**Fig 4:**
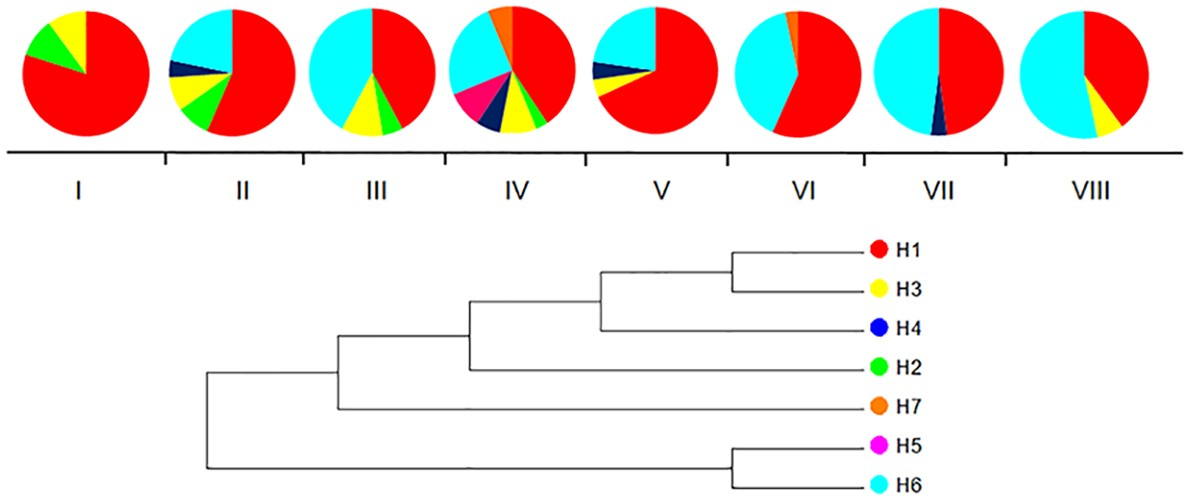
The distribution of haplotypes of rice landraces in eight altitude zones. Phylogenetic relationships among the haplotypes based on a neighbor-joining analysis are indicated below the map. Pie charts show the proportions of haplotypes within each altitude zone. Haplotypes are indicated by different colors.

We examined the geographic distribution of *Ehd1* (Early heading date 1) haplotypes along the altitudinal gradient because this gene had the most polymorphisms. As shown in Fig 4, haplotype H_1 was a major haplotype and was widely distributed across all altitude zones, indicating an ability to adapt to a wide range of conditions in different altitude zones. Haplotype H_6 was a *japonica*-like haplotype; its frequency was 78.08% in *japonica* rice, but only 0.97% in *indica* rice. Interestingly, at altitudes above 1,600 m, we detected an increase in the frequency of haplotype H_6 as the altitude increased. This result suggested that the *japonica*-like haplotype is more adaptable to high-altitude zones than to low-altitude zones.

### Genetic differentiation between and within subpopulations

Hierarchical AMOVA results are presented in Table 2. We found that a significant proportion of the total variance was explained by variance between subpopulations for all gene loci, ranging from 11.33% (*STS22*) to 85.81% (*Os1977*). We observed significant genetic differentiation among altitude zones within subpopulations at most loci, except for *Os1977* and *STS22*. The proportion of the total variance that was explained by variance among altitude zones within subpopulations ranged from 1.63% (*CatA*) to 9.30% (*S5*). We detected significant genetic differentiation within altitude zones for all ten loci, representing 14.00% (*Os1977*) to 89.76% (*STS22*) of the total variance. For all genes, the average genetic variation between subpopulations (46.92%) and within altitude zones (48.98%) was greater than that found among altitude zones within subpopulations (4.10%). These results were further supported by an AMOVA based on SSRs indicating that 61.85% of the total variation was due to variation within altitude zones, 32.53% between subpopulations, and only 5.62% was found among altitude zones within subpopulations.

**Table 2:** Hierarchical analysis of molecular variance for eight altitude zones in two subpopulations (AMOVA).

Source of variation	*CatA*	*GBSSII*	*Os1977*	*STS22*	*STS90*	*S5*	*Pid3*	*Ehd1*	*GS3*	*GS5*	SSRs	Average
												(gene regions)
Between subpopulations	69.59**	40.50**	85.81**	11.33**	12.25**	44.11**	48.73**	71.42**	26.02**	59.43**	32.53*	46.92
Among altitude zones	1.63*	7.37**	0.2	-1.1	7.28**	9.30**	4.51*	2.73*	5.16*	3.92**	5.62*	4.10
within subpopulations												
Within altitude zones	28.78**	52.12**	14.00**	89.76*	80.47**	46.59**	46.76**	25.85**	68.82**	36.65**	61.85*	48.98

*P < 0.05;

**P < 0.01.

Based on *F*_ST_, we observed less genetic differentiation among altitude zones within subpopulations (-0.0282–0.1633 for *indica* rice and -0.0491–0.1178 for *japonica* rice; Table 3) than between subpopulations (0.0395–0.8559) for the genes. For SSRs, we also observed less genetic differentiation among altitude zones within subpopulations (0.0222–0.2415 for *indica* rice and 0.0374–0.2419 for *japonica* rice; Table 3) than between subpopulations (0.3231–0.5335). These results were fairly consistent with the AMOVA results, indicating that there was less genetic differentiation among altitude zones within subpopulations than between subpopulations. The average *F*_ST_ between subpopulations for gene loci (0.4922) was higher than that for SSR loci (0.3893), indicating that genetic differentiation between the *indica* and *japonica* subpopulations might be more obvious in gene regions.

**Table 3:** Pairwise divergence (*F*_ST_) between altitude zones within subpopulations and between subpopulations.

Locus		*F*_ST_ between altitude zones		*F*_ST_ between subpopulations
		*Indica*	*Japonica*	*Indica* vs. *Japonica*
*CatA*	Average	0.054	-0.0074	0.7227
	Min/max	-0.1148/0.3654	-0.1220/0.1559	0.5638/0.8916
*GBSSII*	Average	0.1633	0.0380	0.4458
	Min/max	-0.0790/0.8382	-0.1057/0.3277	0.1255/0.8508
*Os1977*	Average	0.0210	-0.0073	0.8559
	Min/max	-0.0969/0.1716	-0.0666/0.0543	0.7527/0.9665
*STS22*	Average	-0.0069	-0.0491	0.0395
	Min/max	-0.1250/0.1773	-0.1130/0.0212	-0.0208/0.1225
*STS90*	Average	-0.0282	0.1178	0.1613
	Min/max	-0.1172/0.1015	-0.1409/0.4032	-0.0403/0.5385
*S5*	Average	0.1633	0.0525	0.5982
	Min/max	-0.0605/0.7025	0/0.1826	0.2254/1.0000
*Pid3*	Average	-0.0153	0.0929	0.4884
	Min/max	-0.0868/0.1982	-0.0830/0.3744	0.2275/0.7777
*Ehd1*	Average	-0.0113	0.1095	0.6758
	Min/max	-0.0928/0.0786	-0.1269/0.4804	0.3233/0.9764
*GS3*	Average	0.0926	0.0466	0.309
	Min/max	-0.0698/0.4750	-0.0920/0.2188	0.0815/0.5823
*GS5*	Average	-0.0026	0.1034	0.6249
	Min/max	-0.0916/0.2324	-0.1113/0.3784	0.4649/0.7267
SSRs	Average	0.0854	0.1060	0.3893
	Min/max	0.0222/0.2415	0.0374/0.2419	0.3231/0.5335
Average (gene regions)		0.0430	0.0497	0.4922

### Isolation by altitude

**Fig 5:**
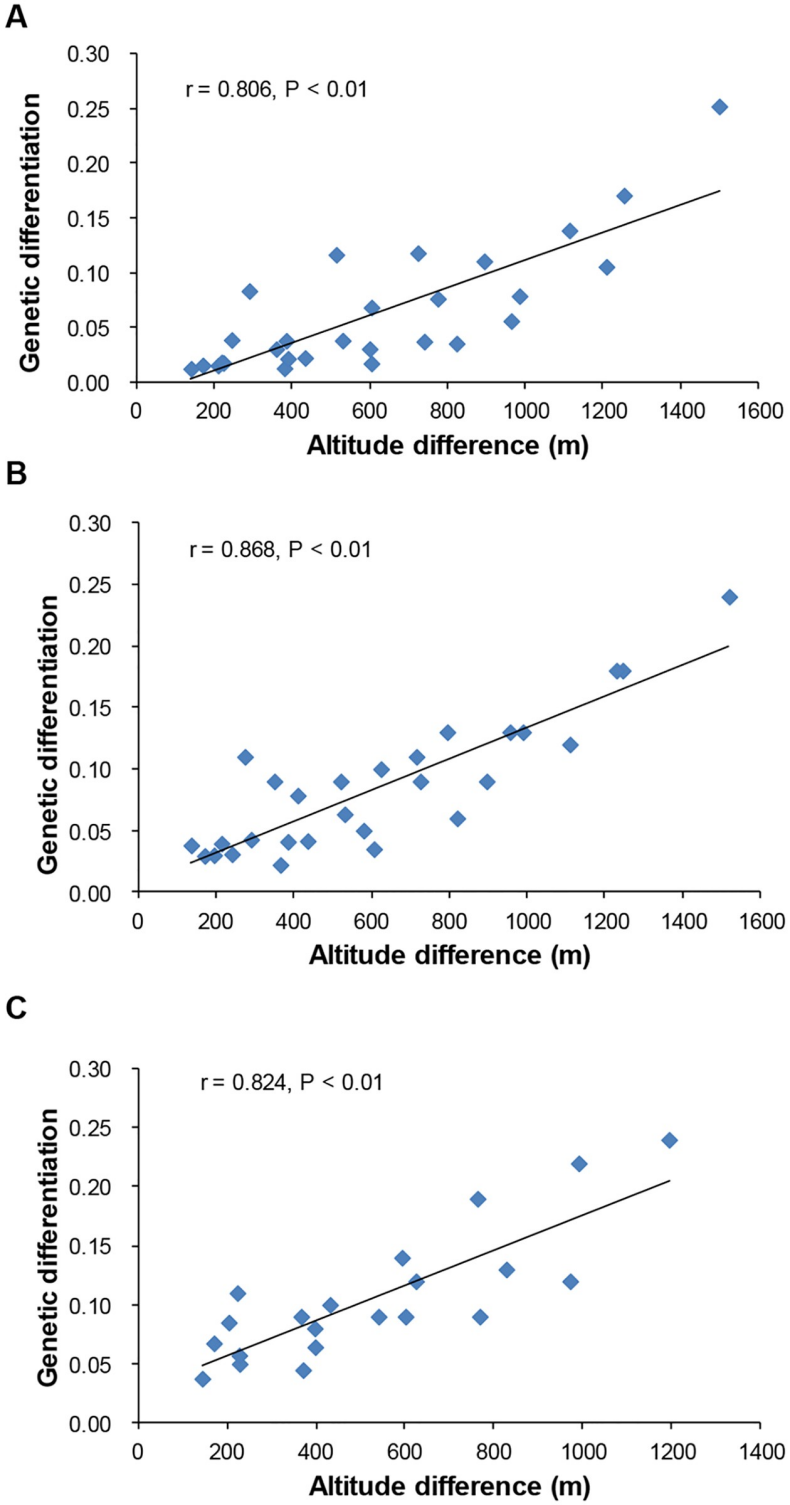
Patterns of isolation by altitude in rice landraces based on SSR markers. (A) Isolation by altitude in the entire population; (B) isolation by altitude in the *indica* subpopulation; (C) isolation by altitude in the *japonica* subpopulation. The correlation coefficients are 0.806, 0.868 and 0.824 for the entire population, *indica* subpopulation and *japonica* subpopulation, respectively.

The structure analysis suggested that genetic variation is altitude-dependent and specifically that there may be an isolation by altitude. We examined the isolation by altitude using a Mantel’s test implemented in Arlequin 3.01 [37] and observed a significant correlation between genetic differentiation and altitude difference (*r* = 0.806, P < 0.01) in the entire population based on SSR data (Fig 5A). Notably, we observed the lowest average genetic differentiation (*F*_ST_) between rice in the middle altitude zone and those in other altitude zones, but we detected higher genetic diversity in middle altitude rice (S6 and S7 Tables). Similarly, we detected significant positive correlations between genetic differentiation and altitude difference both in the *indica* subpopulation (*r* = 0.868, P < 0.01, Fig 5B) and *japonica* subpopulation (*r* = 0.824, P < 0.01, Fig 5C). Furthermore, we found a significant correlation between genetic differentiation and altitude difference for most gene loci in the entire population, except for *STS90* and *Pid3*, with correlation coefficients ranging from 0.510 to 0.819 (Table 4).

**Table 4:** Mantel’s tests for the correlation between genetic differentiation and altitude difference.

	*CatA*	*GBSSII*	*Os1977*	*STS22*	*STS90*	*S5*	*Pid3*	*Ehd1*	*GS3*	*GS5*	SSRs
Total	0.6002**	0.6402**	0.6536**	0.5100*	-0.1141	0.7621**	-0.0833	0.6617**	0.8190**	0.7094**	0.8060**
*Indica*	0.2999	0.2287	-0.1707	-0.0277	-0.2961	0.8768**	0.1234	-0.1297	0.9118**	-0.3439	0.8675**
*Japonica*	0.1949	0.5387*	0.0809	0.3574	-0.1609	0.3425	0.3002	0.0455	0.6588*	0.6837*	0.8240**

*P < 0.05;

**P < 0.01.

## Discussion

### Genetic structure and differentiation of rice landraces

Using a model-based structure analysis and a PCA, we found that the rice landraces from Yunnan were clearly differentiated into *indica* and *japonica* subpopulations. Within each group, the accessions could be further separated into two subgroups according to altitude, i.e., a lower altitude subgroup and higher altitude subgroup, with the exception of a few accessions from middle altitude zones. These results suggested that altitude is an important determinant of population structure. Genetic differentiation between *indica* and *japonica* has been detected based on analyses of morphological traits [39], isozymes [40,41], and DNA markers [42], consistent with our results at the DNA level [7]. Based on the observed distributions across altitudes, *indica* landraces adapt to a wider range of conditions than *japonica* landraces, although both types were cultivated across a wide altitude range above 800 m in Yunnan. We verified the relationship between altitude and the distribution of subspecies in a correlation analysis (S4 Fig); *indica* rice landraces were more frequent at lower altitudes, while *japonica* rice landraces were more frequent at higher altitudes. The observed patterns of distribution were largely attributable to their adaptations to different ecological conditions, especially temperature; the air temperature in the high-altitude zone was significantly lower than that in the low-altitude zone in Yunnan (S5 Fig).

### Genetic diversity and haplotype distribution of rice landraces

Yunnan is a center of diversity for rice in China, and several studies have examined diversity in Yunnan rice landraces. However, to date, such studies focused exclusively on morphological traits or SSR markers [6, 7, 43, 44, 45, 46]. In this study, we used both nuclear gene loci and SSRs to analyze the extent and structure of genetic variation in rice landraces across a wide range of altitudes in Yunnan.

We observed higher levels of nucleotide diversity in both *indica* and *japonica* subpopulations than those reported in a previous survey [12] of 30 Asian cultivated rice accessions based on ten genes (*θ*_π_ = 0.0029, *θ*_w_ = 0.0021 for *indica*; *θ*_π_ = 0.0013, *θ*_w_ = 0.0011 for *japonica*), indicating a high level of nucleotide diversity in rice landraces from Yunnan. Based on SSRs, we detected more genetic variation in *indica* rice than in *japonica* rice, which was in agreement with the results of a survey employing 24 SSR markers to examine genetic diversity of 113 rice varieties from Yunnan [47]. By contrast, Zhang et al. found higher genetic diversity in *japonica* rice landraces than in *indica* rice landraces of Yunnan based on 20 SSRs [6]. The inconsistent results can probably be explained by differences in sample size, number of molecular markers, marker types, and so on.

In a haplotype analysis, we further found obviously genetic differentiation between *indica* and *japonica* rice landraces in some gene regions. Remarkably, for *Ehd1* (Fig 4), which promotes short-day flowering and controls FT-like gene expression [18], the frequency of the *japonica*-like haplotype H_6 increased as the altitude increased above 1,600 m. Indeed, we found that the average days to heading for rice landraces containing haplotype H_6 was less than for landraces containing other haplotypes (S8 Table). These results indicated that early flowering is a critical trait for adaptation to high-altitude zones with short growing seasons and low air temperatures and the *japonica*-like haplotype is more adapted to high-altitude zones [48].

### Isolation by altitude in rice landraces

Rice landraces in Yunnan are planted across a wide range of altitudes [7,8]. A series of environmental factors vary along the altitudinal gradient, and climatic differences could cause genetic divergence among populations [49,50]. High-altitude environmental conditions (e.g., short growing seasons and low temperatures) severely constrain the survival and reproduction of plants and populations; thus, divergence might be due to local adaptation [49,51]. In the present study, we observed a strong association between genetic differentiation and altitude difference at different population levels (Fig 5), indicating an altitude-dependent isolation pattern. Thus, the isolation by altitude was a main factor influencing genetic differentiation among populations in different altitude zones. This raises the question of how isolation by altitude occurred in rice landraces of Yunnan.

The isolation by altitude pattern was consistent with higher rates of gene flow among rice landraces at similar altitudes than along an altitudinal gradient [52]. For inbred cultivated rice, where little to no pollen flow occurs, gene flow must occur by seed movement, and specifically by seed exchange among farmers [2]. Altitude can result in reproductive isolation due to phenological shifts (e.g., flowering period), reducing seed exchange between populations at different altitudes; this may be an important mechanism for creating genetic divergence among populations along the altitudinal gradient [53].

Generally, seeds are exchanged more frequently among farmers within an altitude zone, resulting in high genetic diversity within these zones. Differentiation occurs between altitude zones, reflecting more limited seed exchange. Interestingly, we found that the rice landraces located in the middle altitude zone had the lowest average genetic differentiation (*F*_ST_) with landraces of other altitude zones, but showed higher genetic diversity. These results suggested that seed exchange among farmers in neighboring altitude zones reduces rice genetic structure. In addition, the social structure of communities at different altitudes, such as differences in language or customs, can also influence seed exchange preferences. For example, *Daizu* people dwell in the flatlands or semi-mountainous areas under 1,200 m, while *Lahuzu* people prefer medium or highly mountainous regions [54]. These groups have different cultures and food preferences; the *Daizu* people enjoy *glutinous* rice, but *Lahuzu* people prefer red rice varieties. Hence, seed exchange might be restricted between populations at different altitudes under these circumstances, i.e., language and custom barriers. Overall, our results highlight the influence of isolation by altitude on the pattern of the gene flow and genetic differentiation of rice landraces from mountainous regions.

## Supporting information

S1 Fig Geographic localities of rice landraces sampled in this study (When a figure is similar but not identical to the original image, and is therefore for illustrative purposes only). The localities of rice landraces are indicated by solid circles. Detailed information of the materials is provided in S1 Table.
(PDF)

S2 Fig Schematic diagrams of ten nuclear loci and locations of the sequenced regions. Exons are shown as open boxes and exon numbers are labeled with capital roman numbers. Thin lines between open boxes indicate introns. Locations of primers for each fragment are shown above the diagrams.
(PDF)

S3 Fig The ΔK statistic for each given *k* using 48 SSRs (A) and 89 SNPs from ten genes (B). (PDF)

S4 Fig Correlation between the proportion of *indica* rice and altitude (A) and between the proportion of *japonica* rice and altitude (B). (PDF)

S5 Fig The average of daily maximum (red), average (green) and minimum (blue) air temperatures (°C) at the low (Hekou, 425 m) (A) and high-altitude sites (Xianggelila, 2,274 m) (B) during the period from April to October of 2012–2016. (PDF)

S1 Table List of samples included in the study, including their origin and subpopulation. (PDF)

S2 Table Descriptions of agronomic traits of rice landraces from each altitude zone in Yunnan. (PDF)

S3 Table Summary of SSR markers and primer sequences. (PDF)

S4 Table Summary of sequenced genes and primer sequences used in this study. (PDF)

S5 Table Genetic diversity of model-based populations based on SSRs. (PDF)

S6 Table Genetic diversity of populations in each altitude zone based on SSRs. (PDF)

S7 Table Genetic distances among populations in different altitude zones based on SSRs. (PDF)

S8 Table Days to heading for rice landraces with different haplotypes of *Ehd1*. (PDF)

S1 Dataset Final assembled DNA sequences of *CatA*. (TXT)

S2 Dataset Final assembled DNA sequences of *GBSSII*. (TXT)

S3 Dataset Final assembled DNA sequences of *Os1977*. (TXT)

S4 Dataset Final assembled DNA sequences of *STS22*. (TXT)

S5 Dataset Final assembled DNA sequences of *STS90*. (TXT)

S6 Dataset Final assembled DNA sequences of *S5*. (TXT)

S7 Dataset Final assembled DNA sequences of *Pid3*. (TXT)

S8 Dataset Final assembled DNA sequences of *Ehd1*. (TXT)

S9 Dataset Final assembled DNA sequences of *GS3*. (TXT)

S10 Dataset Final assembled DNA sequences of *GS5*. (TXT)

S11 Dataset Detial information of 48 SSR primer alleles. (XLSX)
