# Supplementary figures and images for: Genetic structure and isolation by altitude in rice landraces of Yunnan, China revealed by nucleotide and microsatellite marker polymorphisms
Source: PLoS One. 2017 Apr 19;12(4):e0175731. doi: 10.1371/journal.pone.0175731 (PMC5396909; doi:10.1371/journal.pone.0175731)

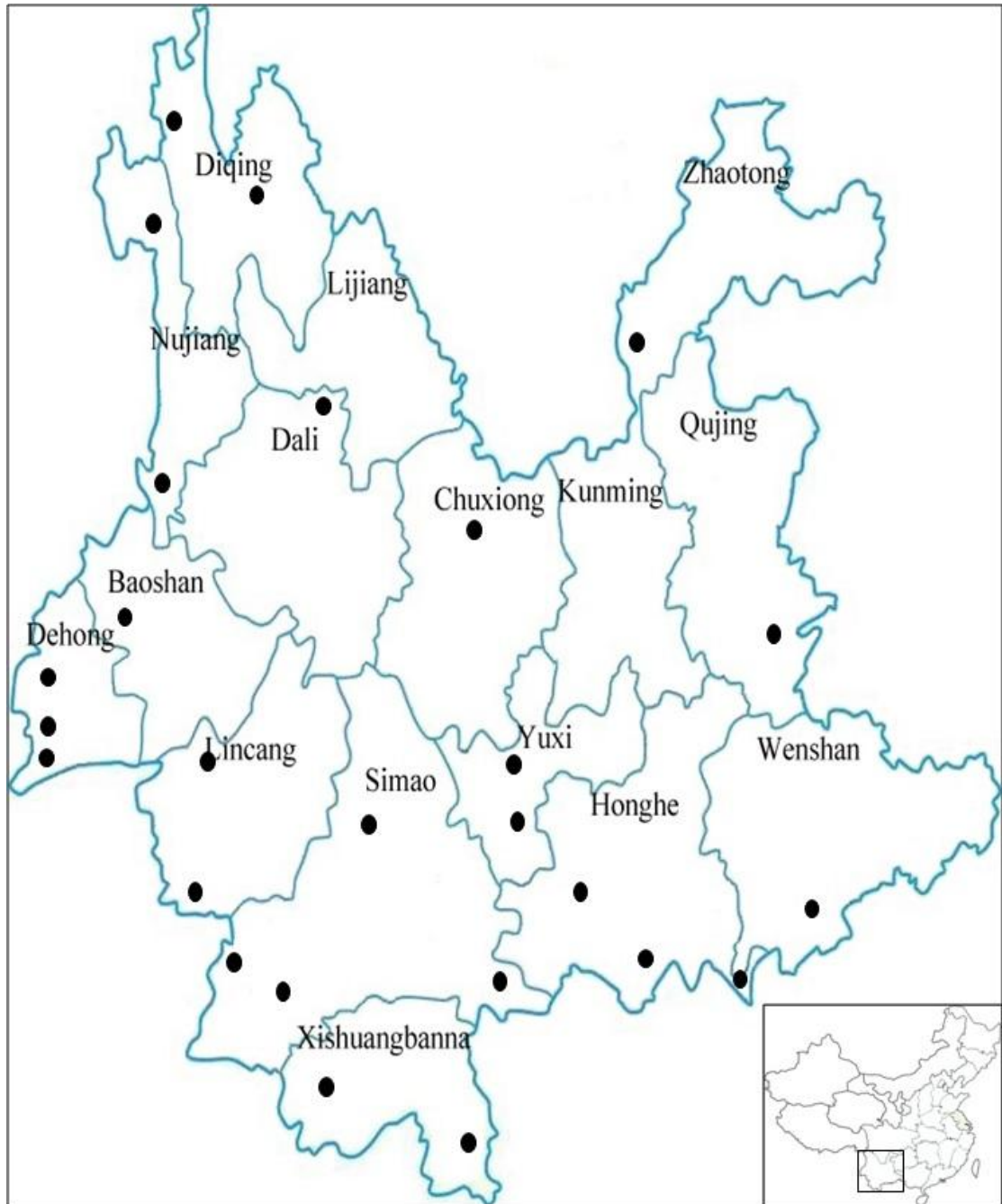

Supplement: S1 Fig — The localities of rice landraces are indicated by solid circles. Detailed information of the materials is provided in S1 Table. (PDF) [file pone.0175731.s001.pdf]

A. *CatA*

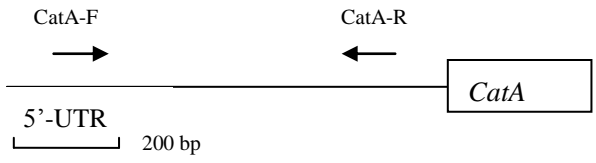

B. *GBSSII*

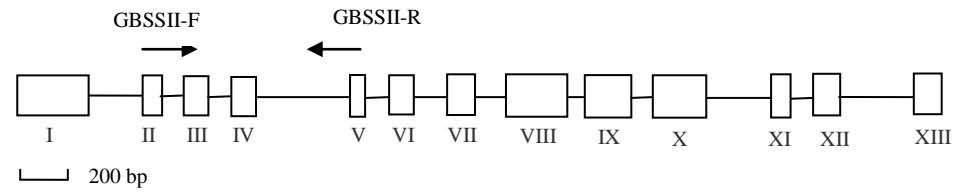

C. *Os1977*

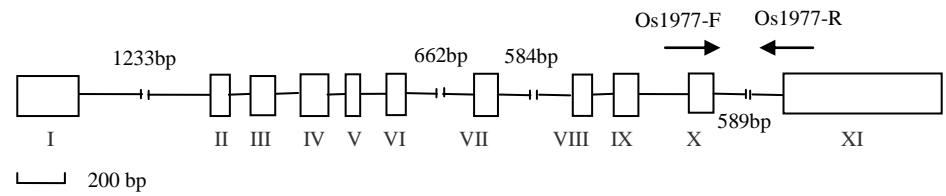

D. *STS22*

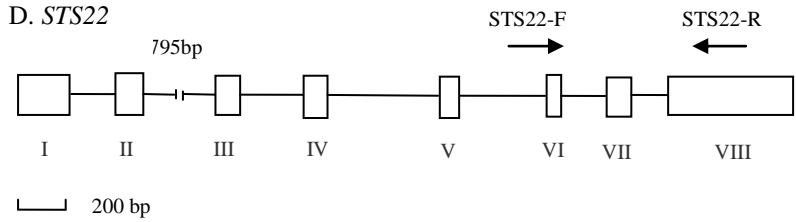

E. *STS90*

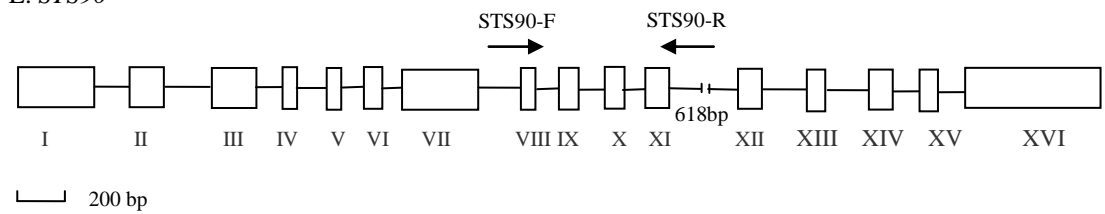

F. *S5*

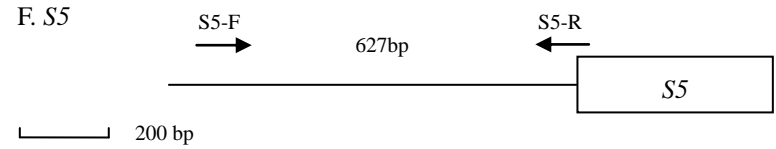

G. *Pid3*

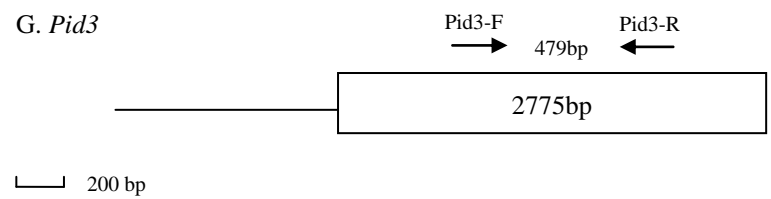

H. *Ehd1*

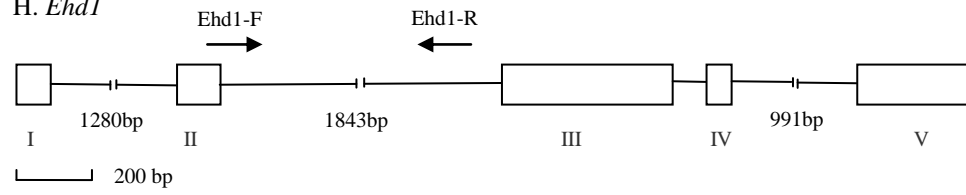

I. *GS3*

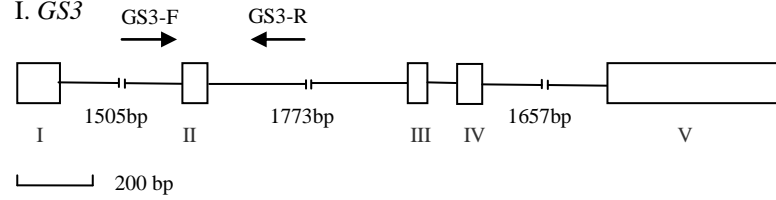

J. *GS5*

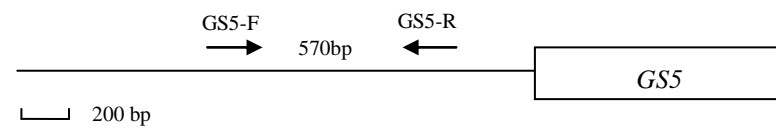

Supplement: S2 Fig — Exons are shown as open boxes and exon numbers are labeled with capital roman numbers. Thin lines between open boxes indicate introns. Locations of primers for each fragment are shown above the diagrams. (PDF) [file pone.0175731.s002.pdf]

**A**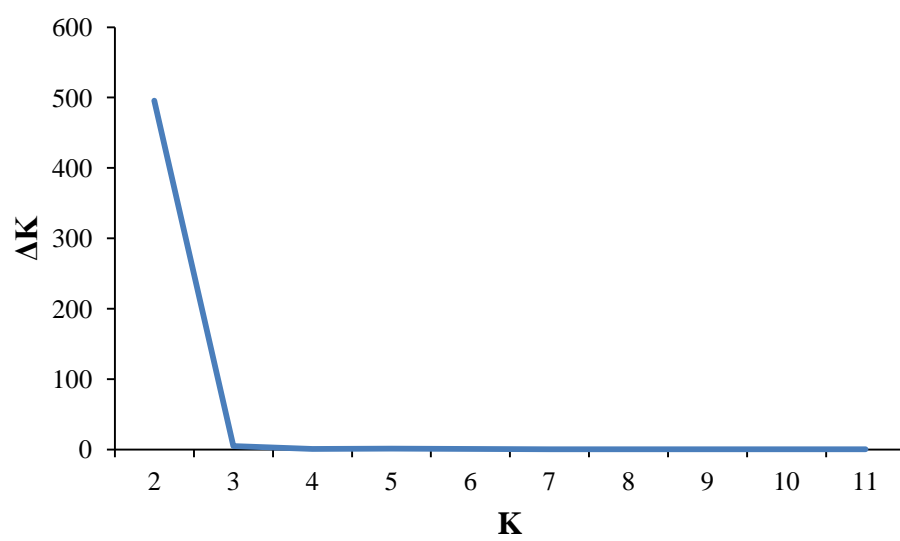**B**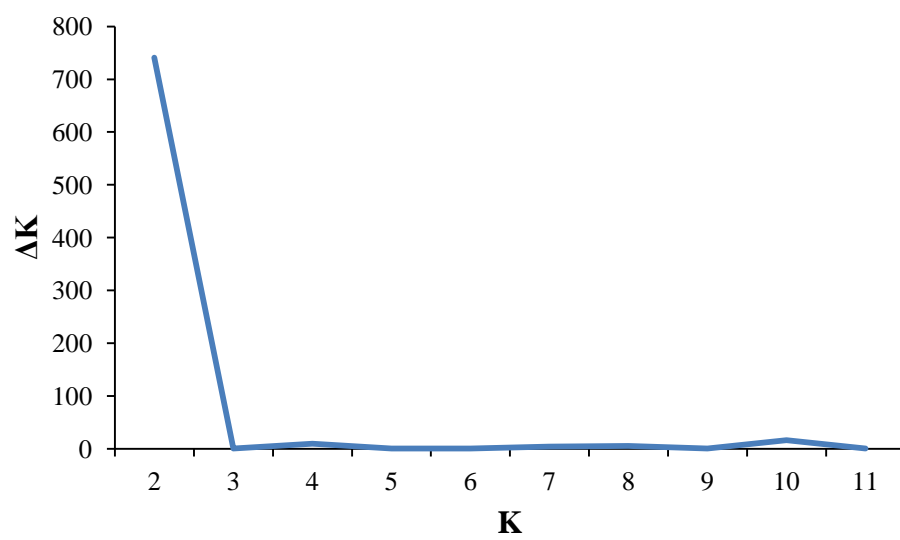

Supplement: S3 Fig — (PDF) [file pone.0175731.s003.pdf]

**A**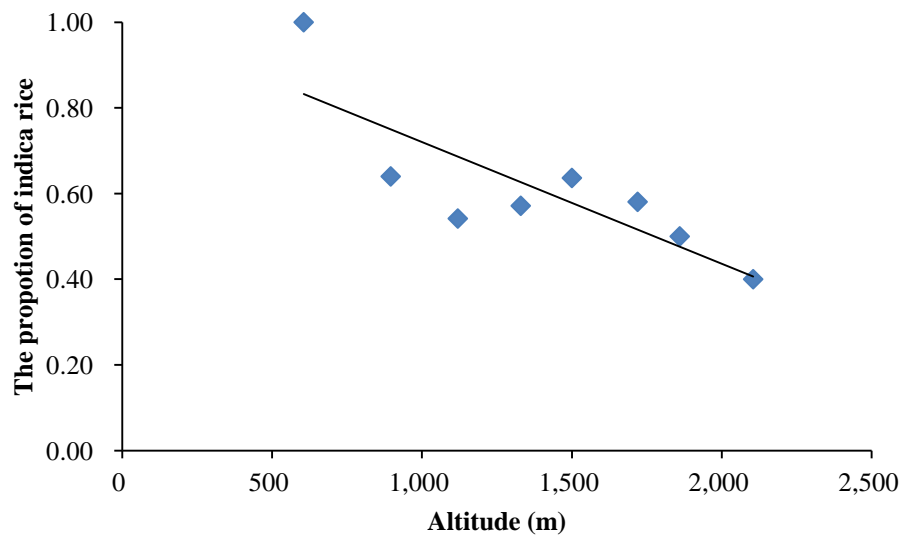**B**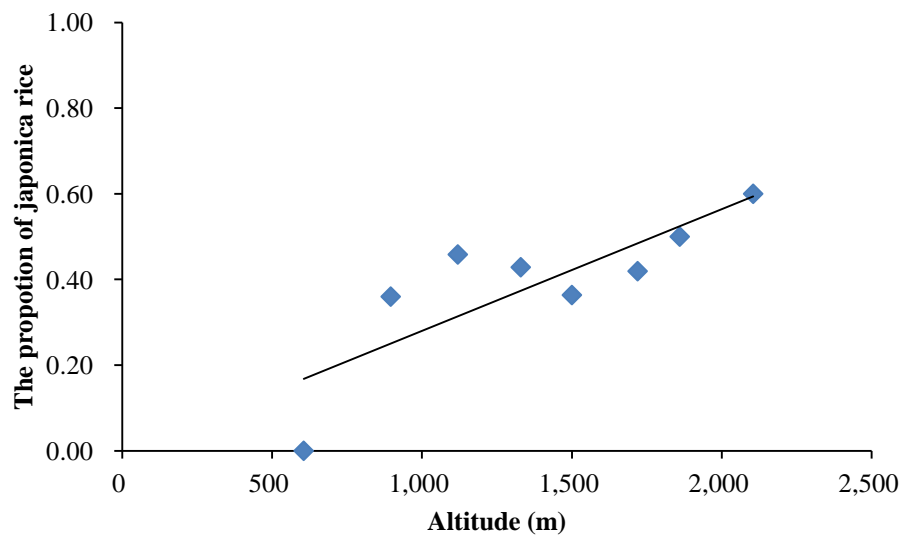

Supplement: S4 Fig — (PDF) [file pone.0175731.s004.pdf]

**A****Hekou (425 m)**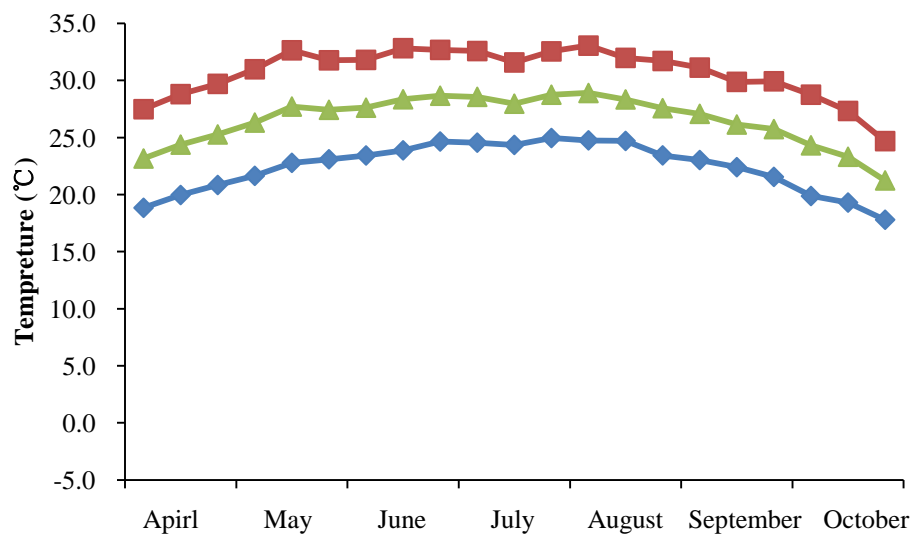**B****Xianggelila (2,274 m)**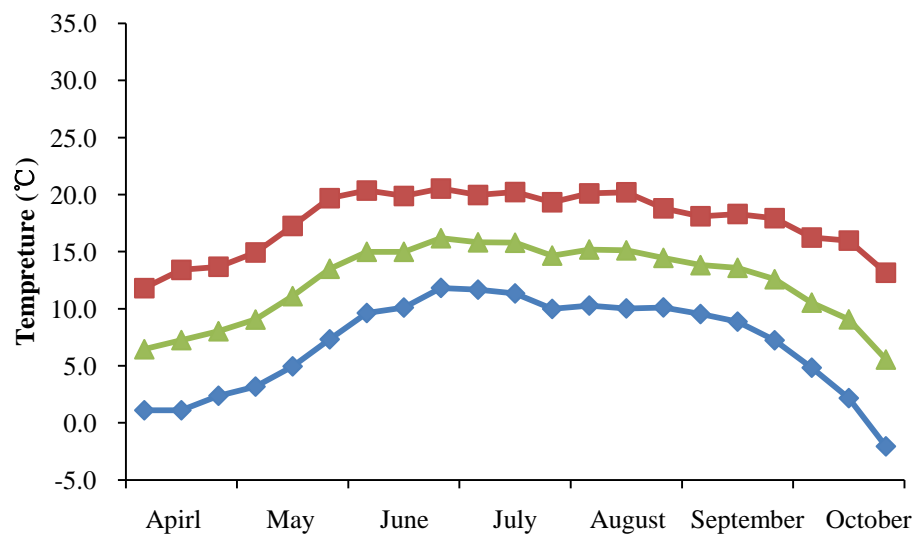

Supplement: S5 Fig — (PDF) [file pone.0175731.s005.pdf]
