# Supplementary material for: Genetic structure and isolation by altitude in rice landraces of Yunnan, China revealed by nucleotide and microsatellite marker polymorphisms
Source: PLoS One. 2017 Apr 19;12(4):e0175731. doi: 10.1371/journal.pone.0175731 (PMC5396909; doi:10.1371/journal.pone.0175731)
Supplement: S1 Table — (PDF) [file pone.0175731.s006.pdf]

No.	Code No.	Accession name	Origin	Subpopulation (SSR marker-based)	Subpopulation components		Altitude (m)	Altitude zones
					Indica	Japonica		
L1	390	Huangpinuo	Lushui,Nujiang	P2	0.001	0.999	2013	Ⅷ
L2	391	Weishangu	Lushui,Nujiang	P1	0.999	0.001	2013	Ⅷ
L3	401	Dabaigu	Gongshan,Nujiang	P1	0.999	0.001	1581	Ⅴ
L4	402	Xiangmi	Xianggelila,Diping	P1	0.999	0.001	2274	Ⅷ
L5	412	Zinuodao	Menghai,Xishuangbanna	P1	1	0	1257	Ⅳ
L6	413	Dalisha	Menghai,Xishuangbanna	P1	1	0	1257	Ⅳ
L7	414	Xiaohuagu	Menghai,Xishuangbanna	P1	0.999	0.001	1257	Ⅳ
L8	415	Jiegunuo	Menghai,Xishuangbanna	P2	0.001	0.999	1300	Ⅳ
L9	419	Manpihongmi	Menghai,Xishuangbanna	P2	0.001	0.999	1353	Ⅳ
L10	420	Manpinuomi	Menghai,Xishuangbanna	P2	0.001	0.999	1353	Ⅳ
L11	421	Manpixianghongnuomi	Menghai,Xishuangbanna	P1	0.999	0.001	1353	Ⅳ
L12	422	Nuogu	Yuanyang,Honghe	P1	0.999	0.001	1087	Ⅲ
L13	423	Zinuogu	Yuanyang,Honghe	P1	1	0	1087	Ⅲ
L14	424	Ganluenimazinuo	Yuanyang,Honghe	P1	0.996	0.004	1425	Ⅴ
L15	425	Yuelianggu	Yuanyang,Honghe	P1	1	0	1656	Ⅵ
L16	426	Zulugu	Yuanyang,Honghe	P1	0.999	0.001	1750	Ⅵ
L17	429	Zinuogu	Yuanyang,Honghe	P1	0.999	0.001	1685	Ⅵ
L18	433	Shuijinghangu	Mengla,Xishuangbanna	P1	0.986	0.014	1371	Ⅳ
L19	434	Baichanggu	Mengla,Xishuangbanna	P1	0.999	0.001	1371	Ⅳ
L20	439	Weibagu	Jiping,Honghe	P1	0.999	0.001	885	Ⅱ
L21	440	Xiaobaigu	Jiping,Honghe	P1	0.969	0.031	885	Ⅱ
L22	441	Dahonggu	Jiping,Honghe	P2	0.003	0.997	885	Ⅱ
L23	442	Huangxiangnuo	Jiping,Honghe	P2	0.002	0.998	972	Ⅱ
L24	443	Liuyuenuo	Jiping,Honghe	P1	0.999	0.001	1370	Ⅳ

L25	444	Daheigu	Yingjiang,Dehong	P2	0	1	1759	Ⅵ
L26	445	Beijinggu	Yingjiang,Dehong	P2	0.001	0.999	1759	Ⅵ
L27	446	Yingjiang 408	Yingjiang,Dehong	P1	0.999	0.001	1240	Ⅳ
L28	451	Xiangnuogu	Yingjiang,Dehong	P1	1	0	872	Ⅱ
L29	454	Liandaogu	Lancang,Simao	P2	0.001	0.999	1378	Ⅳ
L30	455	Dabainuo	Lancang,Simao	P2	0.001	0.999	1378	Ⅳ
L31	463	Dalilandigu	Lancang,Simao	P2	0.001	0.999	1609	Ⅵ
L32	466	Huabanggu "Heidagu"	Tengchong,Baoshan	P2	0.001	0.999	2000	Ⅶ
L33	467	Huabanggu "Baidagu"	Tengchong,Baoshan	P2	0.001	0.999	2000	Ⅶ
L34	469	Huangkenuo	Tengchong,Baoshan	P1	0.999	0.001	1192	Ⅲ
L35	470	Guanzhangnuo	Tengchong,Baoshan	P1	0.999	0.001	1192	Ⅲ
L36	471	Heituannuo	Tengchong,Baoshan	P2	0.001	0.999	1192	Ⅲ
L37	472	Zinuo"Haobixiang"	Tengchong,Baoshan	P1	0.999	0.001	1200	Ⅲ
L38	473	Yelicangnuogu	Tengchong,Baoshan	P1	1	0	1200	Ⅲ
L39	476	Bolinuo	Tengchong,Baoshan	P1	1	0	2137	Ⅷ
L40	478	Aigengnuogu	Longchuan,Dehong	P2	0.001	0.999	1593	Ⅴ
L41	479	Xigu	Longchuan,Dehong	P1	0.999	0.001	1537	Ⅴ
L42	481	Jixuenuo	Longchuan,Dehong	P1	0.998	0.002	911	Ⅱ
L43	482	Hanxiangnuo	Longchuan,Dehong	P1	1	0	1466	Ⅴ
L44	483	Lengshuigu	Longchuan,Dehong	P2	0.001	0.999	1685	Ⅵ
L45	484	Ejinzazixuan	Longchuan,Dehong	P1	0.997	0.003	1685	Ⅵ
L46	485	Yunnanxiaohonggu	Longchuan,Dehong	P1	0.683	0.317	1593	Ⅴ
L47	486	Ledaodun	Longchuan,Dehong	P1	0.988	0.012	911	Ⅱ
L48	488	Nuogu	Ruili,Dehong	P1	0.999	0.001	1358	Ⅳ
L49	490	Laoxianggu	Lushui,Nujiang	P1	0.998	0.002	2013	Ⅷ
L50	492	Shuidao	Gongshan,Nujiang	P2	0.041	0.959	1481	Ⅴ

L51	493	Hongmi	Xianggelila,Diping	P1	1	0	2274	Ⅷ
L52	494	Bayuegu	Xianggelila,Diping	P2	0.001	0.999	2274	Ⅷ
L53	496	Qingkouzinuo	Yuanyang,Honghe	P1	0.999	0.001	1656	Ⅵ
L54	497	Laowotiangu	Mengla,Xishuangbanna	P1	1	0	1371	Ⅳ
L55	498	Fagunuo	Mengla,Xishuangbanna	P2	0.004	0.996	1337	Ⅳ
L56	501	Bayuenuo	Jiping,Honghe	P1	0.918	0.082	885	Ⅱ
L57	502	Lengshuigu	Yingjiang,Dehong	P2	0.012	0.988	1759	Ⅵ
L58	505	Yunnangu	Longchuan,Dehong	P2	0.002	0.998	1537	Ⅴ
L59	506	Honggenxi	Longchuan,Dehong	P1	0.999	0.001	1785	Ⅵ
L60	507	Xueshangu	Longchuan,Dehong	P2	0.002	0.998	1813	Ⅶ
L61	508	Xiaobaigu	Dayao,Chuxiong	P1	0.986	0.014	1168	Ⅲ
L62	509	Wenyilaxiaobaigu	Dayao,Chuxiong	P1	1	0	1988	Ⅶ
L63	510	Honggu	Dayao,Chuxiong	P1	0.999	0.001	1802	Ⅶ
L64	511	Guichao 2	Dayao,Chuxiong	P1	0.985	0.015	1343	Ⅳ
L65	512	Baihebolanggu	Dayao,Chuxiong	P2	0.001	0.999	2022	Ⅷ
L66	513	Baihexiaomagu	Dayao,Chuxiong	P2	0.001	0.999	2022	Ⅷ
L67	514	Eguo	Cangyuan,Lincang	P2	0.001	0.999	1715	Ⅵ
L68	515	Huangkeqitougu	Cangyuan,Lincang	P2	0.001	0.999	1883	Ⅶ
L69	516	Nuogu	Cangyuan,Lincang	P2	0.001	0.999	1883	Ⅶ
L70	517	Menglaixiangmi	Cangyuan,Lincang	P1	0.999	0.001	1281	Ⅳ
L71	518	Lengshuigu	Cangyuan,Lincang	P2	0.001	0.999	1774	Ⅵ
L72	519	Laoshuya	Cangyuan,Lincang	P1	0.999	0.001	1774	Ⅵ
L73	520	Kaolalong	Cangyuan,Lincang	P2	0.001	0.999	1715	Ⅵ
L74	521	Yunxiangnuo	Cangyuan,Lincang	P1	0.991	0.009	732	Ⅰ
L75	522	Lamiangu	Cangyuan,Lincang	P1	0.999	0.001	732	Ⅰ
L76	523	Changmaogu	Cangyuan,Lincang	P2	0.001	0.999	1625	Ⅵ

L77	524	Laoshuya	Cangyuan,Lincang	P1	0.999	0.001	1595	Ⅴ
L78	525	Dabaigu	Cangyuan,Lincang	P1	0.999	0.001	1595	Ⅴ
L79	526	Huangkenuo	Cangyuan,Lincang	P2	0.001	0.999	1595	Ⅴ
L80	527	Haoanlai	Cangyuan,Lincang	P1	0.997	0.003	1449	Ⅴ
L81	530	Nuogu	Yuanjiang,Yuxi	P1	1	0	425	Ⅰ
L82	531	Yidalinuo	Yuanjiang,Yuxi	P1	1	0	601	Ⅰ
L83	532	Xinuogu	Yuanjiang,Yuxi	P1	1	0	601	Ⅰ
L84	533	Honggenggu	Yuanjiang,Yuxi	P1	0.996	0.004	1837	Ⅶ
L85	534	Xiangnuo	Yuanjiang,Yuxi	P2	0.003	0.997	1837	Ⅶ
L86	535	Hongmazhagu	Yuanjiang,Yuxi	P1	1	0	1837	Ⅶ
L87	536	Zigu	Yuanjiang,Yuxi	P1	1	0	1837	Ⅶ
L88	537	Dabainuo	Yuanjiang,Yuxi	P2	0.001	0.999	1837	Ⅶ
L89	538	Laopingu	Yuanjiang,Yuxi	P1	1	0	1812	Ⅶ
L90	539	Gaogengnuo	Yuanjiang,Yuxi	P2	0.001	0.999	1812	Ⅶ
L91	540	Huangkenuo	Yuanjiang,Yuxi	P2	0.001	0.999	1399	Ⅳ
L92	541	Baikenuo	Yuanjiang,Yuxi	P2	0	1	1405	Ⅴ
L93	542	Dengerhaigu	Yuanjiang,Yuxi	P1	0.988	0.012	1400	Ⅳ
L94	543	Dengerhaigu(Hong)	Yuanjiang,Yuxi	P1	0.999	0.001	1400	Ⅳ
L95	544	Duimenshanhonggangu	Yuanjiang,Yuxi	P1	0.999	0.001	1650	Ⅵ
L96	545	Qitougu	Yuanjiang,Yuxi	P1	0.999	0.001	1830	Ⅶ
L97	547	Jiegunuo	Jinggu,Simao	P2	0.003	0.997	1285	Ⅳ
L98	548	Dahuagu	Jinggu,Simao	P1	0.999	0.001	1347	Ⅳ
L99	549	Baihaigu	Jinggu,Simao	P2	0.135	0.865	1455	Ⅴ
L100	550	Xiangnuogu	Jinggu,Simao	P1	1	0	1285	Ⅳ
L101	551	Aijiaobaigu	Jinggu,Simao	P1	1	0	1280	Ⅳ
L102	552	Xiaobaigu	Jinggu,Simao	P1	1	0	1280	Ⅳ

L103	553	Xiaohuangjinnuo	Jinggu,Simao	P2	0.001	0.999	1347	Ⅳ
L104	554	Zigu	Jinggu,Simao	P1	0.999	0.001	1675	Ⅵ
L105	555	Huangkezhainuo	Malipo,Wenshan	P2	0	1	1458	Ⅴ
L106	556	Baikezhainuo	Malipo,Wenshan	P2	0.001	0.999	1458	Ⅴ
L107	557	Honggu	Malipo,Wenshan	P1	1	0	1458	Ⅴ
L108	558	Huinuogu	Malipo,Wenshan	P2	0.003	0.997	1050	Ⅲ
L109	559	Huangnuogu	Malipo,Wenshan	P2	0.001	0.999	1050	Ⅲ
L110	560	Yinzitianzhainuo	Malipo,Wenshan	P2	0.082	0.918	1053	Ⅲ
L111	561	Baigu	Malipo,Wenshan	P1	1	0	832	Ⅱ
L112	562	Dizhainuo	Malipo,Wenshan	P2	0.001	0.999	852	Ⅱ
L113	563	Nuoxuegu	Malipo,Wenshan	P2	0.001	0.999	1050	Ⅲ
L114	564	Zhaizhan	Malipo,Wenshan	P2	0.001	0.999	852	Ⅱ
L115	565	Jiaojizhainuo	Malipo,Wenshan	P2	0.001	0.999	1053	Ⅲ
L116	566	Wuyanuogu	Malipo,Wenshan	P2	0.002	0.998	1050	Ⅲ
L117	567	Babudanuo	Malipo,Wenshan	P1	0.996	0.004	541	Ⅰ
L118	568	Yegaigu	Deqin,Diqing	P2	0.001	0.999	1940	Ⅶ
L119	569	Nuogu	Qiaojia,Zhaotong	P2	0.015	0.985	1169	Ⅲ
L120	570	Honggu	Qiaojia,Zhaotong	P1	0.999	0.001	1169	Ⅲ
L121	582	Xiaobainuo	Jiangcheng,Simao	P1	0.999	0.001	960	Ⅱ
L122	583	Zinuogu	Jiangcheng,Simao	P1	0.992	0.008	948	Ⅱ
L123	584	Xiangnuogu	Jiangcheng,Simao	P1	1	0	872	Ⅱ
L124	585	Mengxinggu	Jiangcheng,Simao	P1	0.999	0.001	872	Ⅱ
L125	586	Xixigu	Jiangcheng,Simao	P1	0.999	0.001	1423	Ⅴ
L126	587	Bendiruangu	Jiangcheng,Simao	P1	0.998	0.002	641	Ⅰ
L127	588	Dadigu	Jiangcheng,Simao	P1	0.945	0.055	641	Ⅰ
L128	589	Jiahenuogu	Jiangcheng,Simao	P1	0.999	0.001	641	Ⅰ

L129	590	Xisanbaizhi	Jiangcheng,Simao	P1	1	0	1023	Ⅲ
L130	591	Manyanggu	Jiangcheng,Simao	P1	0.996	0.004	1063	Ⅲ
L131	592	Cusanbaizi	Jiangcheng,Simao	P1	0.999	0.001	811	Ⅱ
L132	593	Lengshuibaigu	Yongde,Lincang	P1	0.999	0.001	1930	Ⅶ
L133	595	Dabaigu	Yongde,Lincang	P1	0.999	0.001	1650	Ⅵ
L134	596	Changmaonuo	Yongde,Lincang	P1	1	0	1400	Ⅳ
L135	597	Zhazhennuo	Yongde,Lincang	P2	0.001	0.999	837	Ⅱ
L136	598	Daxiangnuo	Yongde,Lincang	P1	1	0	1540	Ⅴ
L137	599	Xiangnuo	Ximeng,Simao	P1	0.999	0.001	994	Ⅱ
L138	600	Zhaluo	Ximeng,Simao	P2	0.001	0.999	994	Ⅱ
L139	601	Ebian	Ximeng,Simao	P2	0.001	0.999	1131	Ⅲ
L140	602	Haonanmao	Ximeng,Simao	P1	0.999	0.001	1053	Ⅲ
L141	603	Malugu	Ximeng,Simao	P1	0.999	0.001	1184	Ⅲ
L142	604	Manhengshuidao	Ximeng,Simao	P1	1	0	1432	Ⅴ
L143	605	Awalaizinuogu	Ximeng,Simao	P1	1	0	1423	Ⅴ
L144	606	Qinuogu	Ximeng,Simao	P1	1	0	1683	Ⅵ
L145	607	Egui-2	Ximeng,Simao	P2	0.001	0.999	1712	Ⅵ
L146	608	Cainuo	Hekou,Honghe	P2	0.035	0.965	956	Ⅱ
L147	609	Baicaigu	Hekou,Honghe	P2	0.001	0.999	893	Ⅱ
L148	610	Huangkenuo	Hekou,Honghe	P1	0.998	0.002	990	Ⅱ
L149	611	Shibanzhainuogu	Hekou,Honghe	P1	1	0	828	Ⅱ
L150	612	Zimi	Hekou,Honghe	P2	0	1	828	Ⅱ
L151	613	Changnuogu	Hekou,Honghe	P1	0.999	0.001	870	Ⅱ
L152	614	Hongbaizajiao	Heqing,Dali	P1	0.999	0.001	1783	Ⅵ
L153	615	Baluchuan	Heqing,Dali	P1	0.999	0.001	1816	Ⅶ
L154	616	Huangpinuo	Heqing,Dali	P2	0.001	0.999	1816	Ⅶ

L155	617	Huangsinuo	Heqing,Dali	P2	0.001	0.999	1816	Ⅶ
L156	618	Zhongyihuangpinuo	Heqing,Dali	P2	0.001	0.999	1783	Ⅵ
L157	619	Xianggu	Heqing,Dali	P1	0.997	0.003	1783	Ⅵ
L158	620	Xiaoxigu	Heqing,Dali	P1	0.838	0.162	1783	Ⅵ
L159	621	Beizigu	Heqing,Dali	P2	0	1	1842	Ⅶ
L160	622	Dahonggu	Heqing,Dali	P1	1	0	1842	Ⅶ
L161	623	Heigu	Heqing,Dali	P2	0.001	0.999	2128	Ⅷ
L162	624	Dashidahonggu	Heqing,Dali	P2	0.001	0.999	2090	Ⅷ
L163	625	Dashimaquepi	Heqing,Dali	P2	0.001	0.999	2090	Ⅷ
L164	626	Heizuibeizigu	Heqing,Dali	P2	0.001	0.999	2090	Ⅷ
L165	627	Dabaigu	Heqing,Dali	P2	0.001	0.999	2090	Ⅷ
L166	628	Nuomi	Xinping,Yuxi	P1	0.998	0.002	1394	Ⅳ
L167	629	Xiangmi	Xinping,Yuxi	P2	0.003	0.997	1394	Ⅳ
L168	630	Daligu	Xinping,Yuxi	P1	0.999	0.001	2041	Ⅷ
L169	631	Honggu	Xinping,Yuxi	P1	0.999	0.001	1779	Ⅵ
L170	632	Laomiuzhainuogu	Xinping,Yuxi	P2	0.001	0.999	1779	Ⅵ
L171	633	Dabaigu	Xinping,Yuxi	P1	0.997	0.003	1918	Ⅶ
L172	634	Honggu	Xinping,Yuxi	P1	0.999	0.001	1745	Ⅵ
L173	635	Yidalinuo	Xinping,Yuxi	P1	0.946	0.054	500	Ⅰ
L174	636	Hongguyangjie	Xinping,Yuxi	P1	0.966	0.034	1809	Ⅶ
L175	637	Nuomi	Xinping,Yuxi	P2	0.001	0.999	1809	Ⅶ
L176	638	Jiegunuo	Xinping,Yuxi	P1	0.991	0.009	1809	Ⅶ
L177	639	Xiaohonggu	Xinping,Yuxi	P1	0.991	0.009	1747	Ⅵ
L178	640	Nuogu	Xinping,Yuxi	P2	0.001	0.999	1747	Ⅵ
L179	641	Laochangzinuomi	Xinping,Yuxi	P1	0.991	0.009	1608	Ⅵ
L180	642	Guogu	Xinping,Yuxi	P1	0.999	0.001	1503	Ⅴ

L181	643	Xiaohuangnuo	Luoping,Qujing	P2	0.05	0.95	1296	Ⅳ
L182	644	Dahuangnuo	Luoping,Qujing	P2	0	1	1296	Ⅳ
L183	645	Bainuogu	Luoping,Qujing	P2	0	1	1151	Ⅲ
L184	646	Shuainuo	Luoping,Qujing	P1	0.999	0.001	1151	Ⅲ
L185	647	Changsuinuogu	Luoping,Qujing	P2	0	1	1151	Ⅲ
L186	648	Erbainuo	Luoping,Qujing	P2	0.001	0.999	1267	Ⅳ
L187	649	Dabaonuo	Luoping,Qujing	P2	0.001	0.999	1267	Ⅳ
L188	650	Beizinuo	Luoping,Qujing	P2	0.001	0.999	1267	Ⅳ
CK1		93-11	typical indica	P1	1	0		
CK2		Nipponbare	typical japonica	P2	0	1		
