# Supplementary material for: Genetic structure and isolation by altitude in rice landraces of Yunnan, China revealed by nucleotide and microsatellite marker polymorphisms
Source: PLoS One. 2017 Apr 19;12(4):e0175731. doi: 10.1371/journal.pone.0175731 (PMC5396909; doi:10.1371/journal.pone.0175731)
Supplement: S2 Table — (PDF) [file pone.0175731.s007.pdf]

Altitude Zone	Location	Total	Days to heading (d)		Plant height (cm)		Effective panicles (NO.)		Grains per panicle (NO.)		Grain length to width (cm)		Spikelet fertility (%)		1,000-grain weight (g)	
			Mean	Range	Mean	Range	Mean	Range	Mean	Range	Mean	Range	Mean	Range	Mean	Range
Ⅰ (< 800 m)	E 99.26-104.70, N 22.59-24.07	10	87	84-95	106.8	98.4-118.6	6.6	5.1-8.6	188.3	143.4-296.1	3.2	2.8-3.8	60.9	27.1-80.7	29.1	19.0-39.0
Ⅱ (800 - 1000 m)	E 97.79-104.70, N 22.54-25.02	25	82	67-92	123.8	91.0-147.7	6.5	3.3-10.5	164	77.7-227.0	2.8	1.9-4.0	76.1	52.0-90.1	27.3	18.0-34.9
Ⅲ (1000 - 1200 m)	E 98.49-104.70, N 22.59-26.91	24	81	66-92	125.4	90.1-164.1	6.5	3.4-9.5	160.6	57.6-265.2	2.5	1.8-3.6	75.9	53.0-90.0	28.3	20.8-38.9
Ⅳ (1200 - 1400 m)	E 97.85-104.31, N 21.46-25.72	35	79	64-91	124	96.1-159.4	6.6	3.0-14.2	152.1	41.9-301.3	2.7	2.0-4.0	73.3	35.1-88.6	28.7	17.3-39.4
Ⅴ (1400 - 1600 m)	E 97.79-104.70, N 22.59-27.74	22	80	64-94	132.6	92.8-165.5	7.1	3.4-9.5	148	32.3-241.8	2.7	1.9-4.3	72.5	47.1-88.1	28.8	21.7-39.5
Ⅵ (1600 - 1800 m)	E 97.79-102.84, N 22.56-26.56	31	77	64-91	125	76.1-171.7	6.9	4.2-9.7	134.3	49.7-234.8	2.8	2.1-3.4	71.9	48.9-93.9	28.8	23.6-36.8
Ⅶ (1800 - 2000 m)	E 97.79-102.01, N 23.15-28.46	26	78	64-92	134.9	88.8-164.8	6.5	3.9-9.8	146.4	56.3-235.0	2.6	2.0-3.2	71.4	51.0-87.8	28.7	22.9-40.8
Ⅷ (<2000 m)	E 98.49-101.99, N 24.07-27.90	15	73	64-88	128	102.3-156.7	7.8	5.9-9.9	112.5	31.6-212.6	2.6	2.2-3.0	65.5	42.1-87.7	26.9	22.6-30.3
