# Supplementary material for: Genetic structure and isolation by altitude in rice landraces of Yunnan, China revealed by nucleotide and microsatellite marker polymorphisms
Source: PLoS One. 2017 Apr 19;12(4):e0175731. doi: 10.1371/journal.pone.0175731 (PMC5396909; doi:10.1371/journal.pone.0175731)
Supplement: S3 Table — (PDF) [file pone.0175731.s008.pdf]

Name	Forward primer	Reverse primer	Chr.No.	Genetic distance (cM)
RM220	ggaaggtaactgtttccaac	gaaatgcttcccacatgtct	1	28.4
RM575	caatttccataggctgcatg	gcttgggttagcgacgac	1	51
RM579	tccgagtggttatgcaaatg	aattgtgtccaatgggctgt	1	61.3
RM488	cagctagggttttgaggctg	tagcaacaaccagcgtatgc	1	102.3
RM521	ttcccttattcctgctctcc	gggatttgcagtgagctagc	2	58.4
RM424	tttgtggctcaccagttgag	tggcgcattcatgtcatc	2	66
RM525	ggcccgtccaagaaatattg	cggtgagacagaatccttacg	2	143.7
RM250	ggttcaaaccaagctgatca	gatgaaggccttccacgcag	2	170.1
RM251	gaatggcaatggcgctag	atgcggttcaagattcgatc	3	79.1
RM282	ctgtgtcgaaaggctgcac	cagtcctgtgttgcagcaag	3	100.6
RM16	cgctagggcagcatctaaa	aacacagcaggtacgcgc	3	131.5
RM504	tctataatgtagcccccccc	tttcaggggcttctaccaac	3	158.6
RM551	agcccagactagcatgattg	gaaggcgagaaggatcacag	4	8.5
RM471	acgcacaagcagatgatgag	gggagaagacgaatgtttgc	4	53.8
RM349	ttgccattcgcgtggaggcg	gtccatcatccctatggtcg	4	146.8
RM280	acacgatccactttgcgc	tgtgtcttgagcagccagg	4	152.3
RM13	tccaacatggcaagagagag	ggtggcattcgattccag	5	28.6
RM593	tcccgtatgtaacgtgcca	gacaagagaacatcgctagg	5	31.4
RM289	ttccatggcacacaagcc	ctgtgcacgaacttccaaag	5	56.7
RM26	gagtcgacgagcggcaga	ctgcgagcgacggtaaca	5	118.8
RM508	ggatagatcatgtgtggggg	acccgtgaaccacaaagaac	6	0
RM586	acctcgcgttattaggtaccc	gagatacgccaacgagatacc	6	7.4
RM584	agaaagtggatcaggaaggc	gatcctgcaggtaaccacac	6	26.2
RM461	gagaccggagagacaactgc	tgatgcggtttgactgctac	6	139.9
RM11	tctcctcttcccccgatc	atagcgggcgaggcttag	7	47
RM560	gcaggaggaacagaatcagc	agcccgtgatacggtgatag	7	54.2
RM10	ttgtcaagaggaggcatcg	cagaatgggaaatgggtcc	7	63.5
RM18	ttccctctcatgagctccat	gagtgcctggcgctgtac	7	90.4
RM38	acgagctctcgatcagccta	tcggtctccatgtcccac	8	28
RM547	taggttggcagaccttttcg	gtcaagatcatcctcgtagcg	8	58.1
RM331	gaaccagaggacaaaaatgc	catcatacatttgcagccag	8	69
RM80	ttgaaggcgctgaaggag	catcaacctcgtcttcaccg	8	97
RM219	cgtcggatgatgtaaagcct	catatcggcattcgcctg	9	11.7
RM409	ccgtctcttgctagggattc	ggggtgttttgctttctctg	9	45.6
RM257	cagttccgagcaagagtactc	ggatcggacgtggcatatg	9	66.1
RM160	agctagcagctatagcttagctggagatcg	tctcatcgccatgcgaggcctc	9	82.4
RM311	tggtagtataggtactaaacat	tcctatacacatacaaacatac	10	25.2
RM258	tgctgtatgtagctcgcacc	tggcctttaaagctgtcgc	10	70.8
RM228	ctggccattagtccttgg	gcttgcggctctgcttac	10	96.3
RM496	gacatgcgaacaacgacatc	gctgcggcgctgttatac	10	113
RM167	gatccagcgtgaggaacacgt	agtccgaccacaaggtgcgttgtc	11	37.5
RM536	tctctcctcttgtttggctc	acacaccaacacgaccacac	11	42
RM209	atatgagttgctgtcgtgcg	caacttgcatcctcccctcc	11	84.7
RM144	tgccctggcgcaaatttgatcc	gctagaggagatcagatggtagtgca	11	123.2
RM20	atcttgtccctgcaggtcat	gaaacagaggcacatttcattg	12	3.2
RM453	cgcatctctctcccttatcg	ctctcctcctcgttgtcgtc	12	28.2
RM309	gtagatcacgcacctttctgg	agaaggcctccggtgaag	12	74.5
RM17	tgccctgttattttcttctctc	ggtgatcctttcccatttca	12	107.4
