# Supplementary material for: Genetic structure and isolation by altitude in rice landraces of Yunnan, China revealed by nucleotide and microsatellite marker polymorphisms
Source: PLoS One. 2017 Apr 19;12(4):e0175731. doi: 10.1371/journal.pone.0175731 (PMC5396909; doi:10.1371/journal.pone.0175731)
Supplement: S4 Table — (PDF) [file pone.0175731.s009.pdf]

Locus	Chr. No.	Aligment length		Primer Sequence (5'-3')	Funtional Association
		Total	Coding		
CatA	2	420	0	TCGTTATCATCAGCGTGGAC<br>CATGCCACTAACTAGCTCAG	Catalase gene
GBSSII	7	420	123	GTGACAGGACAGAAACAGTG<br>TCCAGTGTGCCAGTCATTTG	Granule-bound starch synthase II
Os1977	3	420	0	GTGTTGTTGCTGCATATTTG<br>CCTACGGTCATTCCAAAACC	Porin, eukaryotic type family protein
STS22	4	548	234	GGATGACACTTCACATCCGTCAG<br>CGGAGATTCCCAGGGTAAGTAGG	Imidazoleglycerol-phosphate dehydratase, putative
STS90	12	476	231	TGTTCATCACACTTCTGGGAAGG<br>GTATGGTGCCCATGTGTTCTGG	Phytoene dehydrogenase; hypothetical protein
S5	6	627	0	CGTTCCCTTTCCCTACCTTA<br>TATCTTCTCCGATCCGAGCC	Wide compatibility gene
Pid3	6	479	479	AAGTCCAAATGCACGATATCCTCCG<br>TGACCCAGCAAGACGTAGAAACGTG	Pyricularia oryzae resistance-d3
Ehd1	10	465	0	ATTTGGCAGTGAACATTTGCAG<br>CTTACGGACATTTATGAGGACTCG	Early heading date 1
GS3	3	570	53	ACATGCCCATCTCCCTCGTT<br>GTAGGTGTATGACAGGTTGGACCAG	Major QTL for grain length and weight
GS5	5	570	0	GAAGGATGGAAAGCGAAACTGATT<br>AGTAAGGGCTCATTTGAATCGCAG	Regulator of grain size
