# Supplementary material for: Genetic structure and isolation by altitude in rice landraces of Yunnan, China revealed by nucleotide and microsatellite marker polymorphisms
Source: PLoS One. 2017 Apr 19;12(4):e0175731. doi: 10.1371/journal.pone.0175731 (PMC5396909; doi:10.1371/journal.pone.0175731)
Supplement: S5 Table — (PDF) [file pone.0175731.s010.pdf]

POPs	Allele No.	Genotype No.	Private allele No.	Heterozygosity	Gene diversity	PIC
P1 (*Incia*)	7.6875	10.4375	3.3000	0.0418	0.6166	0.5796
P2 (*Japonica*)	7.7500	10.1458	3.4000	0.0648	0.5736	0.5442
