# Supplementary material for: Genetic structure and isolation by altitude in rice landraces of Yunnan, China revealed by nucleotide and microsatellite marker polymorphisms
Source: PLoS One. 2017 Apr 19;12(4):e0175731. doi: 10.1371/journal.pone.0175731 (PMC5396909; doi:10.1371/journal.pone.0175731)
Supplement: S6 Table — (PDF) [file pone.0175731.s011.pdf]

	Altitude zones	SampleSize	Alleles/locus	Gene Diversity	PIC	Private allelles
I	<800	10	3.48	0.5239	0.4709	3
II	800-1000	25	7.52	0.7056	0.6706	14
III	1000-1200	24	7.58	0.7217	0.6824	7
IV	1200-1400	35	8.44	0.7352	0.6996	19
V	1400-1600	22	7.83	0.7236	0.6875	8
VI	1600-1800	31	8.50	0.7184	0.6808	17
VII	1800-2000	26	7.31	0.6926	0.6476	7
VIII	>2000	15	5.10	0.6316	0.5798	5
