# Supplementary material for: Genetic structure and isolation by altitude in rice landraces of Yunnan, China revealed by nucleotide and microsatellite marker polymorphisms
Source: PLoS One. 2017 Apr 19;12(4):e0175731. doi: 10.1371/journal.pone.0175731 (PMC5396909; doi:10.1371/journal.pone.0175731)
Supplement: S7 Table — (PDF) [file pone.0175731.s012.pdf]

Altitude zone	I	II	III	IV	V	VI	VII
I							
II	0.2186						
III	0.3213	0.1015					
IV	0.3319	0.1088	0.0963				
V	0.3056	0.1035	0.0981	0.0979			
VI	0.3978	0.1501	0.1454	0.1074	0.1059		
VII	0.4937	0.2063	0.1567	0.1535	0.1355	0.0761	
VIII	0.7722	0.3527	0.2741	0.2640	0.2397	0.1411	0.1400
