# Supplementary material for: Genetic structure and isolation by altitude in rice landraces of Yunnan, China revealed by nucleotide and microsatellite marker polymorphisms
Source: PLoS One. 2017 Apr 19;12(4):e0175731. doi: 10.1371/journal.pone.0175731 (PMC5396909; doi:10.1371/journal.pone.0175731)
Supplement: S8 Table — (PDF) [file pone.0175731.s013.pdf]

Haplotype	Days to heading (d)
H1	82.4
H2	83.6
H3	86.4
H4	81.8
H5	78.7
H6	71.3
H7	78.0
